# Adapting and testing a brief intervention to reduce maternal anxiety during pregnancy (ACORN): study protocol for a randomised controlled trial

**DOI:** 10.1186/s13063-016-1274-8

**Published:** 2016-03-22

**Authors:** Esther L. Wilkinson, Heather A. O’Mahen, Pasco Fearon, Sarah Halligan, Dorothy X. King, Geva Greenfield, Jacqueline Dunkley-Bent, Jennifer Ericksen, Jeannette Milgrom, Paul G. Ramchandani

**Affiliations:** Centre for Mental Health, Imperial College London, 7th Floor Commonwealth Building, Hammersmith Campus, Du Cane Road, London, W12 0NN UK; Central and North West London NHS Foundation Trust, Stephenson House, 75 Hampstead Road, London, NW1 2PL UK; Institute of Psychiatry, Psychology & Neuroscience, King’s College London, 16 De Crespigny Park, London, SE5 8AF UK; University of Exeter, Mood Disorders Research Centre, Perry Road, Exeter, EX4 4QG UK; Research Department of Clinical, Educational and Health Psychology, University College London, 1-19 Torrington Place, London, WC1E 7HB UK; Department of Psychology, 2 South, University of Bath, Bath, BA2 7AY UK; School of Public Health, Faculty of Medicine, Imperial College London, Reynolds Building, Charing Cross Campus, St Dunstan’s Road, London, W6 8RP UK; NHS England, Nursing Directorate, Skipton House, 80 London Road, London, SE1 6LH UK; Parent-Infant Research Institute, Department of Clinical & Health Psychology, Centaur Building, Heidelberg Repatriation Hospital, Austin Health, 300 Waterdale Road, Heidelberg Heights, Melbourne, VIC Australia; Psychological Sciences, Level 12, Redmond Barry Building 115, University of Melbourne, Parkville, VIC 3010 Australia

**Keywords:** Antenatal, Anxiety, Group intervention, Cognitive behavioural therapy, Feasibility

## Abstract

**Background:**

National guidelines in the UK, United States of America, Canada, and Australia have recently stressed the importance of identifying and treating antenatal anxiety and depression. However, there is little research into the most effective and acceptable ways of helping women manage their symptoms of anxiety and stress during pregnancy. Research indicates the necessity to consider the unique needs and concerns of perinatal populations to ensure treatment engagement, highlighting the need to develop specialised treatments which could be integrated within routine antenatal healthcare services. This trial aims to develop a brief intervention for antenatal anxiety, with a focus on embedding the delivery of the treatment within routine antenatal care.

**Methods/Design:**

This study is a two-phase feasibility trial. In phase 1 we will develop and pilot a brief intervention for antenatal anxiety, blended with group support, to be led by midwives. This intervention will draw on cognitive behavioural principles and wider learning from existing interventions that have been used to reduce anxiety in expectant mothers. The intervention will then be tested in a pilot randomised controlled trial in phase 2. The following outcomes will be assessed: (1) number of participants meeting eligibility criteria, (2) number of participants consenting to the study, (3) number of participants randomised, (4) number of sessions completed by those in the intervention arm, and (5) number of participants completing the post-intervention outcome measures. Secondary outcomes comprise: detailed feedback on acceptability, which will guide further development of the intervention; and outcome data on symptoms of maternal and paternal anxiety and depression, maternal quality of life, quality of couple relationship, mother-child bonding, infant temperament and infant sleep.

**Discussion:**

The study will provide important data to inform the design of a future full-scale randomised controlled trial of a brief intervention for anxiety during pregnancy. This will include information on its acceptability and feasibility regarding implementation within current antenatal services, which will inform whether ultimately this provision could be rolled out widely in healthcare settings.

**Trial registration:**

Current Controlled Trials ISRCTN95282830. Registered on 29 October 2014.

**Electronic supplementary material:**

The online version of this article (doi:10.1186/s13063-016-1274-8) contains supplementary material, which is available to authorized users.

## Background

Clinically significant levels of anxiety symptoms are experienced by up to half of all women during pregnancy [1], with a high proportion suffering diagnosed anxiety disorders [2–4]; prevalence estimates for generalised anxiety disorder in pregnancy range from 0 to 10.5 % [4]. Furthermore, antenatal anxiety is a strong predictor of postnatal anxiety and depression [3, 5, 6]. There is now a substantial body of evidence from observational cohort studies demonstrating that elevated anxiety in mothers during pregnancy is also associated with an increased risk of a range of short- and long-term adverse outcomes in their children [7] and poorer obstetric outcomes [8]. These include higher rates of preterm delivery [8, 9], and effects on child outcomes such as difficult temperament [10, 11], increased sleep problems [12], poorer cognitive functioning [13], and higher rates of emotional and behavioural problems in early childhood that often persist into later development [7].

National guidelines in the UK [14], the United States of America [15, 16], Canada [17], and Australia [18–20] have recently stressed the crucial importance of identifying and offering treatment for antenatal anxiety and depression. These guidelines have recommended screening for all women as a routine part of antenatal care, along with timely access to appropriate services for assessment and psychological intervention in pregnancy [14–20]. These recommendations were made despite the lack of evidence to guide the direction of treatment for women who have anxiety during pregnancy and the absence of systematic research examining the impact of treating antenatal anxiety on maternal and infant outcomes. This missing link is critical, as the potential opportunity for preventive intervention is large, given the substantial neural, cognitive and socio-emotional developments that occur in the fetal period and the first years of life [7], and potential for recurrent problems in the mother.

It is, therefore, crucial that effective and cost-effective treatments are developed for antenatal anxiety. Recent research strongly suggests that perinatal populations have unique concerns, and that considering the specific needs of perinatal populations is essential to ensure treatment engagement [21–23]. Any treatment for antenatal anxiety must, therefore, be acceptable and relevant to pregnant women, addressing their specific anxieties and unique circumstances and needs during this period. Cognitive Behavioural Therapy (CBT) has been subject to rigorous evaluation and has a strong evidence base for the effective treatment of anxiety disorders [24], including in a guided self-help format [25], but a specific literature investigating the utility of CBT approaches for treating anxiety during pregnancy is largely lacking at present [26, 27]. One of very few interventions that have been further developed and evaluated for antenatal mood difficulties is *Towards Parenthood* [28]. This is a guided self-help intervention designed to help expectant parents prepare for parenthood, which has been developed by Milgrom, Ericksen and colleagues in Australia. This has been tested in a clinical trial [28] and showed promising results, with significant reductions in symptoms of both anxiety and depression at postnatal follow-up compared to routine care.

Recent research has also highlighted the impact that partners have on child development and family functioning [29], and the importance of social support during pregnancy, since this is associated with lower maternal antenatal and postnatal depression and anxiety [6, 30, 31]. More recent policy documents have indicated that pregnancy is an important opportunity to engage partners [32] and have recommended the inclusion of fathers in care and intervention from the antenatal period [33], suggesting that interventions for maternal mental health in the perinatal period should also consider the potential inclusion of partners.

The advantages of intervening in the antenatal period to reduce maternal anxiety are threefold: first, the psychological treatment is delivered in a context in which mothers are already experiencing a high level of contact with healthcare services and frequently attend routine antenatal classes; second, antenatal treatment could reduce the possible negative effects of antenatal anxiety on fetal development; and third, given the associations between antenatal anxiety and postnatal depression and anxiety [3, 5, 6], antenatal intervention offers the potential to prevent some episodes of postnatal depression and anxiety and so to improve maternal wellbeing, as well as reduce the developmental risk to the child of exposure to maternal postnatal depression and anxiety.

Our research proposes the development and testing of an intervention tailored specifically for use with pregnant women experiencing antenatal anxiety, based on CBT principles and incorporating key learning from the *Towards Parenthood* team, for use in a group format led by midwives, in the context of UK antenatal services. Embedding the intervention in routine antenatal care in a brief midwife-led group format means that it has the potential to be a cost-effective mode of delivery and one that is robust to changing healthcare environments, requiring less time and input from midwives than an individual psychological approach and being widely available to women experiencing difficulties with anxiety. It therefore has the potential to improve outcomes for a wide range of pregnant women and their infants, thereby reducing the overall population burden of anxiety during pregnancy. Given the existing national guidelines, the results of the proposed trial could be crucial in informing the development of clinical services.

### Aims and objectives

Following the Medical Research Council guidelines for developing and testing complex interventions [34, 35], the purpose of this research is to assess the feasibility and acceptability of delivering a midwife-led group intervention to reduce levels of anxiety in pregnant women. The objective of phase 1 is to develop an intervention, based on the principles outlined above, for use in the UK in a group setting led by midwives. The objective of phase 2 is to conduct a pilot randomised controlled trial (RCT) comparing the group intervention with treatment as usual. Outcomes will assess: the feasibility and acceptability of recruitment and data collection methods; the acceptability of intervention materials and delivery; likely recruitment and retention rates; and estimates of the range of effect sizes. We will use this information to inform a fully powered RCT.

## Methods/Design

### Study design

The study consists of two phases. Phase 1 is a development stage in which a brief intervention for anxiety will be developed, adapted for pregnancy, drawing on core CBT principles and learning from the team that developed the *Towards Parenthood* intervention [28]. We will develop an intervention for delivery in group format, to be led by midwives and embedded into antenatal care in the UK. In an initial pilot group we will gather detailed feedback about the intervention content and delivery, and will use this information to inform further treatment adaptations. A mixed-methodology design will be used. If a manualised intervention can be successfully developed and delivered to a pilot group in a way that is acceptable to them, and/or modified to take any key suggestions and concerns into account, we will continue to phase 2.

In phase 2, we will conduct a pilot RCT comparing the group intervention for antenatal anxiety (intervention group) with treatment as usual (control group). This protocol follows Consolidated Standards of Reporting Trials (CONSORT) [36] and Standard Protocol Items: Recommendations for Interventional Trials (SPIRIT) [37] (Additional file 1) guidelines for reporting clinical trial protocols.

### Study setting

Participants will be recruited through UK National Health Service (NHS) antenatal scanning clinics at their 12-week scan in London and the South West of England. The intervention will be delivered to participants in these locations as a part of antenatal services.

### Participant inclusion criteria

Eligible participants will be pregnant women who have not had any children, entering their second trimester, aged 18 and over. Women will be invited to participate if they score in the top quartile of scores on the Generalised Anxiety Disorder 7-item scale (GAD-7) [38] at screening. The GAD-7 is a 7-item self-report screening measure for anxiety symptoms, which is widely-used in clinical practice and research.

Partners of women who consent to take part in the study will also be invited to participate. Fully informed consent will be obtained from potential participants for each stage of the research.

### Participant exclusion criteria

Potential participants will be excluded if: they have insufficient understanding of English to complete the intervention or outcome measures; they have a significant illness or disability that would make it difficult for them to participate.

### Recruitment procedure

**Fig. 1:**
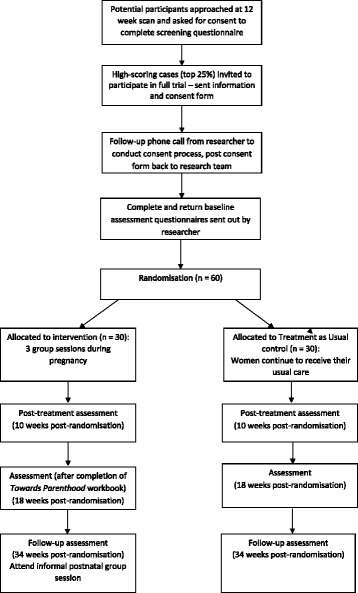
Consolidated Standards of Reporting Trials (CONSORT) flow diagram of progress through the study

After completing the screening questionnaire for anxiety (GAD-7; [38]) and a small number of demographic questions in the antenatal scanning clinic, women who meet eligibility criteria will be sent information on the full study and a consent form. A researcher will follow up with a phone call and, if the individual is willing to take part, will guide them through the consent form. The participant will be directed to send back the signed consent form via mail or online, after which the researcher will provide the participant with the option to complete the baseline questionnaires via mail or online. Upon completion and receipt of the baseline measures, participants will be randomised (see Fig. 1). Women’s partners who agree to participate in the study will be consented separately.

All participants will be offered a £10 voucher at three time points in the study as an expression of gratitude for their participation and will be reimbursed any travel expenses incurred.

### Randomisation

In phase 2, participants will be randomly allocated on a 1:1 basis to either the intervention group or the treatment as usual group using a sealed envelope method, managed by a staff member in a separate research group. Participants will be informed of their group allocation by a researcher over the telephone.

### Sample size

In line with guidelines for pilot RCTs, no formal power calculation has been conducted [39]. A sample size of 30 participants per arm is recommended for feasibility studies as providing sufficient data to gain an accurate estimation of the feasibility and acceptability of the intervention and trial methods [40], and to provide a reasonable range of estimates of the sample size required for a definitive RCT [41]. Thus, 30 participants will receive the intervention (in four groups of approximately 7–8 participants each, plus participants’ partners where possible) and 30 will receive treatment as usual.

### Intervention

The research will adapt and build on existing interventions utilising CBT principles. One such intervention, *Towards Parenthood*, is a workbook-based, psychoeducational intervention that focuses on making the transition into parenthood and on the emotional, social and psychological issues that can arise during pregnancy and early parenthood. The *Towards Parenthood* intervention aims to help expectant parents develop effective coping and parenting skills. In addition, it provides specific information about anxiety, stress and mood problems and incorporates fundamental elements of CBT, which addresses maladaptive cognitions and behaviours. In a clinical trial in Australia [28] it was shown to be effective in reducing symptoms of anxiety (*d* = 0.58), stress (*d* = 0.59) and depression (*d* = 0.6) in women at postnatal follow-up. The workbook incorporates nine sections and women in the Australian trial additionally received weekly telephone support from trained psychologists or postgraduate psychology trainees.

The planned intervention will be developed for optimal efficacy, feasibility and acceptability with a broad population of pregnant women experiencing a range of anxiety symptoms of differing severity, with the aim of reducing the overall population burden of antenatal anxiety.

The proposed format will be a manualised brief intervention, blended with group support. We plan to deliver three group sessions in the antenatal period, led by a trained midwife and psychology supporter, with each session lasting approximately 90 minutes. Sessions will be held at 3-week intervals, which is designed to maintain participant engagement but also allow participants enough time to try out practical strategies between sessions. As a further measure to support participants and maintain engagement, the midwife and psychologist delivering the groups will also send a weekly email to each participant to remind them to practise the skills from the previous group session and to ask whether they have any questions about the material. This can be stepped up to a telephone call if there is no response from a participant or if a telephone call is requested by the participant. Telephone calls will be limited to a maximum of 15 minutes in length, unless clinical imperative dictates otherwise.

**Fig. 2:**
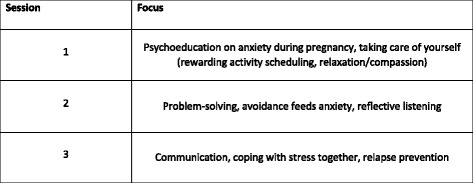
Focus of midwife-led group support sessions

The three group sessions are designed to address specific antenatal anxieties, and will support participants in developing key practical skills. A group mode of delivery has strong potential to be more cost-effective and sustainable and less time-consuming for midwives than individual support, and will also enable mutual support and interaction between group members, thereby fitting well with the existing models of antenatal care. Furthermore, it could be easily integrated into current routine NHS antenatal services and would, therefore, be widely available and accessible to a broad range of pregnant women. The specific parameters of the intervention and its delivery will be finalised in light of the pilot work undertaken in phase 1 of the research programme, but the aim is to base the three group sessions on a number of key themes, including psychoeducation on anxiety during pregnancy, problem-solving, communication and other strategies and techniques to help with managing anxiety and stress including self-care and compassion (Fig. 2).

In the first two sessions, participants will be split into separate groups for part of the session: one group for pregnant women and the other for partners. This will allow pregnant women to work on specific skills to help with their anxiety, whilst partners will work on skills and approaches to support their pregnant partner. The remainder of each session, and the entire third session, will be delivered to the group as a whole, to allow couples to develop coping strategies together. At the end of the three group sessions support moves to be more self-led and participants will be given the full *Towards Parenthood* workbook to work through. There will also be a follow-up session for participants at approximately 8 weeks postnatally. This will be an informal session which will give participants a chance to meet again and to check in with the midwives.

All women in the intervention arm will continue to receive their usual care during pregnancy, and will have access to the usually available range of interventions for antenatal anxiety and other physical and mental health problems.

### Control: treatment as usual

Women randomised to the control group will continue to receive their usual care for antenatal anxiety. Although there is currently no standard model of care for antenatal anxiety, this may include care provided by their general practitioner (GP), midwife, health visitor, local Improving Access to Psychological Therapies (IAPT) service or community mental health team. The care received by women in the trial will be actively monitored by the research group, using the Adult Service Use Scale (AD-SUS).

### Assessments and outcome measures

#### Feasibility and acceptability

The main study outcomes will assess the feasibility and acceptability of the intervention and study design. The primary outcome will be feasibility of recruitment and data collection, examined through assessing the following: (1) number of participants meeting eligibility criteria, (2) number of participants consenting to the study, (3) number of participants randomised, (4) number of sessions completed by those in the intervention arm, and (5) number of participants completing the post-intervention outcome measures. Furthermore, we will record reasons for study withdrawal, non-participation and non-attendance of intervention sessions.

Detailed feedback will be gathered from all participants and the midwife and psychologist delivering the intervention, in order to assess the acceptability of the intervention and trial methods. Feedback will be gained from participants allocated to the intervention arm through brief questionnaires at the end of each group session and a more detailed post-intervention feedback questionnaire. Semi-structured acceptability interviews will be conducted with a purposeful sample of participants from both study arms at post-intervention assessment. Open-ended questions will be asked about participants’ impressions of the intervention and group delivery; the relevance and suitability of the intervention content; perceived benefit of the intervention; problems or difficulties experienced with the sessions, and recommendations for changes or developments to the intervention; their experience of being a part of the study; and their impressions of current routine antenatal care. Interviews are designed to last approximately 30–60 minutes and will be conducted face-to-face. Qualitative data analysis will be conducted following the interviews.

Fidelity to the intervention manual will be examined using video and audio tapes of intervention sessions. A random sample of 10 % of therapy tapes will be assessed for fidelity by two of the investigators.

#### Data collection

At each assessment point, questionnaires will either be posted to participants and returned via post, or participants will be provided with a link to a website where they can complete the measures online. Measures will be collected at baseline, post intervention (10 weeks post randomisation), 8 weeks post intervention (18 weeks post randomisation), and postnatal follow-up (34 weeks post randomisation); the measures of mother-child relationship, infant temperament and infant sleep will be collected at postnatal follow-up only. Partners will also complete measures of anxiety, mood and quality of couple relationship at each assessment point.

#### Clinical outcome measurements

A range of clinical outcome measures will be included in order to assess the feasibility and acceptability of these measures, and estimates of levels of symptoms in both trial arms at each assessment time point. Measures of the following will be included:

1. Maternal anxiety, using the GAD-7 [38], a 7-item scale measuring symptoms of generalised anxiety disorder. The GAD-7 has excellent internal consistency (Cronbach’s *α* = .92) and good test-retest reliability (intraclass correlation = 0.83) [38]. When used in perinatal populations the GAD-7 has yielded a sensitivity of 61.3 % and specificity of 72.7 %, using a cut-off score of 13 [42]
2. Maternal mood, using the Edinburgh Postnatal Depression Scale (EPDS) [43], a 10-item self-report scale used to assess antenatal and postnatal depression [43–45]. The EPDS has a high level of test-retest reliability (intraclass correlation = 0.92) [46]. In the second trimester of pregnancy the sensitivity of the EPDS is estimated to be 88 % and the specificity 91 %, when using a cut-off score of 9/10 [44]
3. Pregnancy-specific worries, using the 10-item pregnancy-related anxiety scale [47]. The pregnancy-related anxiety scale has acceptable internal reliability (Cronbach’s *α* = .78) [47]
4. Couple relationship, using the Dyadic Adjustment Scale (DAS) [48], a 32-item self-report measure of relationship adjustment. The DAS has been reported to have excellent internal consistency (Cronbach’s *α* = .915) [49]
5. Birth outcomes, using an 8-item maternal self-report questionnaire, developed for this study
6. Mother-child relationship, using the 25-item Postpartum Bonding Questionnaire (PBQ) [50]. The PBQ has been demonstrated to have acceptable internal consistency (Cronbach’s *α* = .76) [51]. For identification of any disorder of the mother-infant relationship the PBQ has yielded a specificity of 74 % and sensitivity of 84 % [52]
7. Infant temperament, using a modified version of the Infant Behaviour Questionnaire (IBQ) [53]. Each subscale of the IBQ has been reported to have acceptable or good internal reliability: activity level (*α* = .73), smiling and laughter (*α* = .85), fear (*α* = .80), distress to limitations (*α* = .84), soothability (*α* = .84), and duration of orienting (*α* = .72) [53]
8. Infant sleep, using the Brief Infant Sleep Questionnaire [54]. This questionnaire has good test-retest reliability; significant correlations are reported between the repeated sleep measures for daytime sleep duration (*r* = .89), settling time (*r* = .94), nocturnal sleep-onset time (*r* = .95), nocturnal sleep duration (*r* = .82), number of night wakings (*r* = .88), and duration of nocturnal wakefulness (*r* = .95) [54]
9. Health-related quality of life of the mother, using the EuroQol-5D-3 L measure [55] recommended by National Institute for Health and Care Excellence (NICE) for inclusion in economic evaluations
10.  Health economics: the economic component of the proposed work will involve use of the Adult Service Use Schedule (AD-SUS) [56], a questionnaire designed for application to mental health populations, including depression and anxiety

### Data analysis

#### Quantitative data analysis

Data analysis will be primarily descriptive to aid the planning of a future RCT. Participant flow through the study will be presented following CONSORT guidelines [36]. Descriptive data will be presented in the form of means and standard deviations; medians and ranges; or percentages with 95 % confidence intervals, as appropriate depending on the data being described. The following will be calculated: (1) percentage of participants meeting eligibility criteria, (2) percentage of individuals consenting to the study, (3) percentage entering the randomisation phase, (4) the number of sessions completed by those in the treatment arm, (5) percentage completing the outcome measures at post-treatment follow-up, (6) between group pre-post effect sizes and confidence intervals on the GAD-7 and EPDS.

#### Qualitative data analysis

Qualitative interviews following the treatment groups will be transcribed verbatim. Transcripts will be analysed in detail using thematic analysis [57], informed by grounded theory methods. Atlas® software will be used to assist in organising content related to themes.

### Data management

Data will be stored in line with NHS ethical procedures and a standard protocol is in place to outline data storage and security procedures. Participants’ questionnaires will be coded with a numerical identifier and kept separately from any identifiable personal information such as names or addresses. Data quality will be checked using double data entry for 10 % of the data and standard data management protocols will be followed.

### Safety monitoring and reporting

A standard protocol is in place to deal with situations in which a participant reports risk to themselves or others. This protocol outlines the response of the researcher and necessary steps to take in order to ensure safety of the participant and others and to establish contact with caregivers or emergency support. If there are concerns about a child’s safety, a standard protocol for dealing with child protection issues will be followed. All risk or safety issues will be reported to the principal investigator, who will take any necessary further steps and provide on-going clinical supervision to members of the research team. The same protocol will be followed should participants disclose any information which is concerning to the research team, or if they score highly (at risk) on the questionnaire assessing mood (EPDS) (Additional file 2).

Debriefing sessions and clinical supervision with the individuals delivering the intervention will take place regularly.

If a participant’s condition should worsen or a co-morbid condition become apparent during the course of the trial the study team would refer the participant to existing healthcare services for additional treatment. In line with ethical guidelines, participants will be informed that should they wish to discontinue with the intervention they can withdraw at any point. If further care for any participant is needed post trial, the study team will refer the participant to existing healthcare services.

Any serious adverse events will be recorded and reported to the sponsor, the hosting NHS trust and to the ethics committee as appropriate.

### Ethical approval

The trial received ethical approval from the London – Riverside National Research Ethics Service on 15 April 2014 (Research Ethics Committee reference number: 14/LO/0339). The trial will be conducted in accordance with the Data Protection Act at all times. The participants will be identified by a study specific participant number in all databases. Names and any other identifying detail will not be included in any study data electronic file. Where sample sizes are very small extra care will be taken to ensure that individual participants cannot be identified from the research data.

### Dissemination

A range of dissemination strategies will be used:

1. A summary report and presentations on study findings will be prepared for clinical services, particularly primary care GP surgeries and maternity services. A presentation of findings will also be planned for clinical conferences and the annual Clinical Research Network conference
2. The trial team will present findings at relevant national and international conferences, and will submit papers on the feasibility RCT results and acceptability interviews to peer-reviewed journals. A full study report will be submitted to the National Institute for Health Research (NIHR)
3. The Patient and Public Involvement advisory group will advise and support on dissemination to service users. This will be achieved through our existing networks, which include close collaborations with national parenting websites, and through further contacts developed with this project
4. Newsletters and a summary of results will be prepared for stakeholder organisations, including those on the trial advisory group. Media releases and talks to non-specialist audiences will also be planned
5. Regular newsletters and a final summary report of trial findings will be sent to all study participants

## Discussion

This two-phase feasibility study is designed to assess questions of feasibility and acceptability, in order to inform the design of a potential future substantive RCT. Detailed acceptability feedback will inform the further development of the intervention.

This group intervention led by midwives has the potential to be an acceptable, effective and cost-effective intervention for anxiety and stress during pregnancy. If effective it could improve short- and long-term outcomes for both mother and child, and reduce the overall population burden of anxiety during pregnancy. It is designed to fit alongside existing antenatal services and could, therefore, potentially be rolled out widely across the NHS.

### Trial status

Recruitment is ongoing, and began in November 2014.

## Additional files

Additional file 1: SPIRIT Checklist. (PDF 130 kb) Additional file 2: Standard Operating Procedure where participant has elevanted levels of depressive symptoms. (PDF 386 kb)

## Abbreviations

AD-SUS: Adult Service Use Schedule
CBT: Cognitive Behavioural Therapy
DAS: Dyadic Adjustment Scale
EPDS: Edinburgh Postnatal Depression Scale
GAD-7: Generalised Anxiety Disorder 7-item scale
GP: general practitioner
IAPT: Improving Access to Psychological Therapies
IBQ: Infant Behaviour Questionnaire
NHS: National Health Service
NIHR: National Institute for Health Research
PBQ: Postpartum Bonding Questionnaire
RCT: randomised controlled trial
