# Supplementary material for: Adapting and testing a brief intervention to reduce maternal anxiety during pregnancy (ACORN): study protocol for a randomised controlled trial
Source: Trials. 2016 Mar 22;17:156. doi: 10.1186/s13063-016-1274-8 (PMC4804571; doi:10.1186/s13063-016-1274-8)
Supplement: Additional file 2: — Standard Operating Procedure where participant has elevanted levels of depressive symptoms. (PDF 386 kb) [file 13063_2016_1274_MOESM2_ESM.pdf]

Standard Operating Procedure for identifying participants with high levels of depressive symptoms and those at risk of harm

1. INTRODUCTION

The ACORN study involves assessing levels of depression and anxiety with self-reported questionnaire measures. It is possible that a participant may score highly on these questionnaires, indicating low mood and potential risk to themselves (Edinburgh Postnatal Depression Scale, EPDS). A participant may also report potential risk to themselves in a group session or interview with a midwife or researcher. In the occurrence of these cases, the midwife or researcher has a duty of care for the participant and should follow procedures to ensure that the participant has access to support. The ACORN study child safeguarding standard operating procedure should be followed in the event that risk to a participant's child is identified.

2. PURPOSE

This standard operating procedure outlines the response of the researchers and midwives and necessary steps to be followed, should the identification of participants with high levels of depressive or anxious symptoms, or concerns about potential risk to a participant or other person's safety occur. This is in order to ensure safety of the participant and others, and to establish contact with caregivers, GPs, or emergency support.

3. RESPONSIBILITIES

3.1 Researchers

Researchers should be vigilant when assessing self-reported questionnaire measures and in interviews with participants. Should clinically significant scores on these questionnaires or potential risk to a participant or other person be identified, the researcher should be sensitive when discussing this with the participant, and ensure that the participant is made aware of the support available, and that any concerns are reported to the principal investigator and participant's GP (thereby informing a member of their clinical care team). Copies of letters sent to GP's should also be sent to the respective participant. Researchers should follow steps outlined in the risk procedure flowchart below. Any serious adverse events are also to be recorded and reported to the host site (Imperial Healthcare NHS Trust), and the sponsor NHS Trust (CNWL NHS Foundation Trust) and to the ethics committee as appropriate.

3.2 Midwives

Midwives should be vigilant during group sessions and individual discussions with participants. Should any information be disclosed indicating low mood or potential risk to a participant or other person during these times the midwife should be sensitive when discussing this with the participant, and ensure that the participant is made aware of the support available, and that any concerns are reported to the principal investigator. Midwives should follow steps outlined in the risk procedure flowchart below.

3.3 Supervisors

Supervisors should take any necessary further steps to provide support and on-going clinical supervision to members of the research team should such a situation occur.

RISK PROCEDURE FLOWCHART

Start here

EPDS score > 12
Or scores 1-3 on Q10

Thoughts of suicide or self-harm indicated in interview/group session/any other source

EPDS score ≤ 12
and scores 0 on Q10

Report to and discuss with Principal Investigator, or substitute senior clinician

No further action to be taken.

Telephone participant and complete Risk of Suicide Assessment (Appendix A) and/or other questions as appropriate (guidance outlined in the 'discussing high scores' SOP)

If unable to contact participant discuss with principal investigator or substitute senior clinician and send a letter to the participant. A letter should not be send to the GP without a participant's consent unless a medium or high risk level is indicated.

Discuss with Principal Investigator or substitute senior clinician using all available information.
Determine risk level

Risk Level: Low
Inform GP by letter. Advise participant to make an appointment with GP.*

Risk Level: Medium
Inform GP by telephone and letter. Advise participant to make an appointment with GP.*

Risk Level:
High (actively suicidal)
Advise participant that immediate help will be arranged.*

ACTION TO TAKE IN THE CASE OF IMMEDIATE RISK:

Do not leave participant alone, or if on telephone, do not hang up.

Involve supervisory clinician right away - contact principal investigator of study (Dr Paul Ramchandani) or substitute senior clinican, should the principal investigator not be available. Then (with clinician if possible) follow the chain of contact below:

1. GP / out of hours GP; if not
2. Crisis team; if not
3. Clinician accompanies to A&E; if not (or interview is over telephone)
4. Call ambulance.

Any serious adverse events should be recorded and reported to the host site (Imperial Healthcare NHS Trust), the sponsor NHS Trust (Central and North West London NHS Foundation Trust) and to the ethics committee as appropriate.

West London Mental Health NHS Trust
Crisis Resolution Team – Hammersmith & Fulham: **020 7386 1146**
24 hour support line: **0300 1234 244**
A & E:
Charing Cross Hospital, St Dunstan's Road, W6 8RF
St Mary's Hospital, South Wharf Road, W2 1NY

Appendix A – Risk of Suicide Assessment

If a participant answers 1-3 on question 10 on the Edinburgh Postnatal Depression Scale (EPDS), or if risk of suicide or self-harm is identified during clinical interview, the following protocol should be followed by researchers or midwives facilitating group sessions.

"I see that you've said / you mentioned that……. These are thoughts / feelings that people suffering from low mood often have, but it's important to make sure you are receiving the right kind of support. So if it's OK, I would now like to ask you some more questions that will explore these feelings in a little more depth."

Thoughts

1. As I mentioned, often people who are experiencing low mood or depression have thoughts/feelings of harming themselves or ending their lives. Do you ever have thoughts/feelings of harming yourself?	Yes/No<br>if **yes** - details
2. Do you ever have any thoughts/feelings of ending your life?	Yes/No<br>If **yes** - details

Plans

3. Do you know how you would end your life?	Yes/No<br>If **yes** – details
4. Have you made any actual plans to end your life?	Yes/No<br>If **yes** – details

Actions

5. Have you made any actual preparations to end your life?	Yes/No<br>If **yes** – details
6. Have you ever attempted suicide in the past?	Yes/No<br>If **yes** – details

Prevention

7. Is there anything stopping you from harming yourself or ending your life at the moment?	Yes/No<br>If **yes** – details
8. Do you feel that there is any immediate danger that you will harm yourself or end your life?	Yes/No<br>If **yes –** details
